# Knowledge, usability and challenges of e-learning platforms for continuing Professional Development of healthcare professionals at University Teaching Hospital of Kigali

**DOI:** 10.1186/s12909-024-05585-x

**Published:** 2024-06-03

**Authors:** Agnes Mukamana, Jean Claude Byungura, Felix Manirakiza, Gerard Rushingabigwi

**Affiliations:** 1 Health Informatics Department, Regional Centre of Excellence in Biomedical Engineering and e-Health (CEBE), University of Rwanda, Kigali, Rwanda; 2 Department of Business Information Technology, College of Business and Economics (CBE), University of Rwanda, Kigali, Rwanda; 3 Pathology Department, College of Medicine and Health Sciences, University of Rwanda, P.O. Box 3286, Kigali, Rwanda; 4 Pathology Department, University Teaching Hospital of Kigali, P.o. Box 655, Kigali, Rwanda; 5 Biomedical Engineering Program, Regional Centre of Excellence in Biomedical Engineering and e-Health (CEBE), University of Rwanda, Kigali, Rwanda

**Keywords:** E-learning usability, Healthcare professionals, CPD

## Abstract

**Background:**

Healthcare professionals constitute a critical component of clinical care services. To provide the expected service, they must continuously develop their profession through continuous learning. This kind of learning is recognized as continuing professional development (CPD). Traditionally, CPD is offered onsite. Onsite training is associated with some barriers that prevent healthcare professionals from attending such educational activities, including financial difficulties and long distance. This is why online learning is proposed to overcome these barriers.

**Objective:**

The main purpose was to evaluate usability, knowledge and challenges of e-learning platforms for CPD of healthcare professionals at University Teaching Hospital of Kigali (CHUK).

**Methods:**

The cross-sectional quantitative study approach was utilized; the data was collected at the workplace of nurses, midwives, and allied health professionals by using a pre-designed questionnaire. The data were analyzed using Statistical Package for the Social Sciences (SPSS) version 25 and presented as frequencies.

**Results:**

A significant majority was aware of CPD e-learning platforms. For example, 95.7% of the participants were familiar with these platforms, indicating that they had some degree of knowledge about their existence and purpose. Regarding the mode of accessing CPD courses, 82.1% of participants preferred online platforms, demonstrating a strong will to use e-learning platforms.

**Conclusion:**

This study highlighted a high level of awareness and utilization of CPD e-learning platforms among healthcare professionals at CHUK, additionally, participants expressed confidence in using the platforms but emphasized the need for further support and training.

## Introduction

The field of Continuing Professional Development (CPD) in healthcare has a rich historical background, dating back centuries within the medical field and institutionalized teaching in the Royal Medical Colleges. In the 20th century, CPD gained global recognition as a formal approach to assist healthcare professionals in acquiring the necessary knowledge and skills for their professional growth [1].

In the UK, the US and Australia, CPD has been mandated for nurses and allied healthcare professionals to maintain competence and provide evidence-informed care. However, a lack of comprehensive data exists regarding the global adoption of CPD requirements and their impact on the skills and knowledge development of healthcare provider [2]. The core concept of CPD emphasizes lifelong learning and continuous improvement, with a focus on enhancing professional competence and personal performance [3].

In Sub-Saharan Africa, CPD has proven to be particularly vital in updating the knowledge, skills, and practices of healthcare professionals, especially in fields such as tuberculosis (TB) and Human Immunodeficiency Virus (HIV) treatment and diagnosis. Online education platforms have shown promise in offering flexible and efficient education in this regard [4].

However, the cost of attending, travel distances, family responsibilities, understaffing, and the inability to take time off from work have prevented many healthcare professionals from participating in these training programs. Online learning holds the potential to overcome these barriers by providing a world-class education to anyone, anywhere, and anytime as long as they have internet connectivity [5–8].

Rwanda has been offering Continuing Medical Education (CME) through yearly sessions organized by the faculty of medicine of the former National University of Rwanda (NUR) and the Rwanda Medical Association (RMA) since 1995. The Ministry of Health (MoH) and Rwanda Biomedical Center (RBC) also provide continuing training to healthcare professionals, especially those working in public health facilities [9–11].

In Rwanda, several online CPD providers offer e-learning opportunities for healthcare professionals. There is a critical knowledge gap in understanding the usability and challenges associated with the use of e-learning platforms for CPD within healthcare settings among healthcare. Despite the potential benefits of e-learning, it remains unclear how well healthcare professionals at University Teaching Hospital of Kigali (CHUK) are adapting to and utilizing these online platforms. Existing research has not extensively explored the actual usage patterns, satisfaction levels, and barriers faced by healthcare professionals in engaging with e-learning for their professional development. In-depth insights into knowledge, usability and challenges are essential to ensure the effectiveness of e-learning in improving the skills and knowledge of healthcare professionals.

Therefore, we conducted this study to assess healthcare provider’s knowledge about CPD e-learning platforms, their usability and the challenges they face in adopting these platforms for CPD. The findings of this research are expected to provide valuable insights in the field of healthcare education and have the potential to drive positive and innovative changes by effectively integrating digital solutions for e-learning into the continuing professional education landscape for healthcare professionals in Rwanda.

## Methods

### Study area and design

**Fig. 1:**
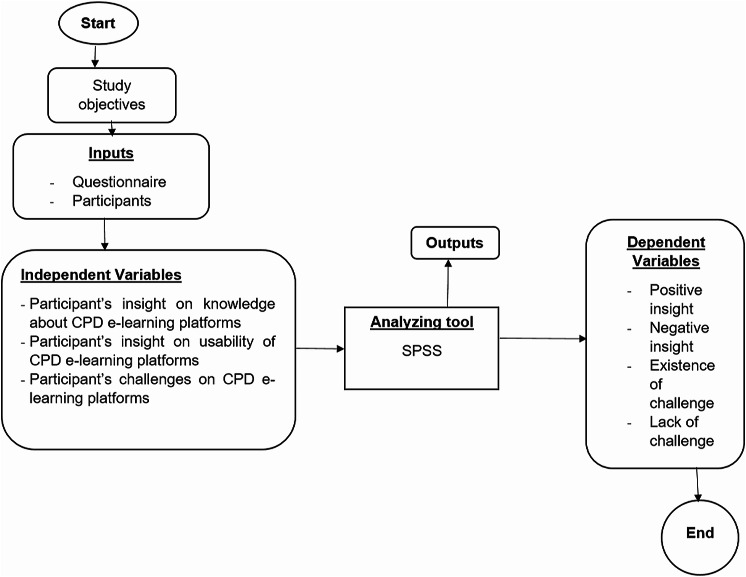
Theoretical framework of the study

This study employed a descriptive cross-sectional design and was conducted among healthcare professionals, specifically nurses, midwives and allied health professionals working at University Teaching Hospital of Kigali (CHUK) who have previously used CPD e-learning platforms in their CPD courses. We chose this study design due to the exploratory nature of our research and its specific contextual focus on the Rwandan health sector and the current CPD e-learning platforms used by healthcare professionals. The theoretical framework for our study is presented in Fig. 1.

### Study site and population

The research was conducted at CHUK, located in Kigali city, Nyarugenge district, Rwanda. CHUK is one of the largest referral hospitals in the country, providing quality healthcare to the population. It is actively involved in clinical research, healthcare professionals training, and offers technical support to district hospitals. The hospital offers a wide range of clinical services and specialties.

These include allied health sciences, surgery, accident and emergency, internal medicine, mental health, anesthesiology and critical care, gynecology and obstetrics, pediatrics, maternal and neonatology, Ear, Nose &Throat (ENT), ophthalmology, neurosurgery, pediatric surgery, urology and dermatology.

The study was conducted in different departments to be able to have insight from people of different skills and different healthcare responsibility.

The study population comprised of nurses, midwives, and allied health professionals who are currently working at CHUK and who were using Rwandan CPD e-learning platforms in their courses. Healthcare professionals unavailable during the data collection period in March 2023 or unwilling to participate in the study were excluded.

### Sample size and sampling strategy

In this research, the Taro Yamane’s simplified formula [12] was used to calculate and determine the sufficient and representative sample size. For this study, the entire study population was of 597 subjects (i.e., nurses, midwives, and allied health professionals who are working at CHUK). Using Taro Yamane’s formula, we can determine the sample size (n) based on the total research population (N) and the margin of error (e). The formula is n = N / [1 + N*(e)^2].

Then we sought a confidence level of 95%; therefore, our estimated error (e) was 0.05.

The calculated sample size was 597/ [1 + 597(0.05) 2] = 240. Therefore, we administered research questionnaires to 240 participants but ultimately, Among the total, at least 140 participants completed the survey questionnaire.

In this study, we used convenience sampling techniques, one type of non-probability sampling strategies whereby the study sample is drawn from the entire targeted population depending on their close suitability and availability for the research [13].

Convenience sampling involved selecting participants who were readily available and willing to participate, considering the limited time due to their busy work schedules. Another criterion was that the participants should have at least used one of the Rwandan CPD e-learning platforms for their CPD training.

### Data collection

In this research, primary data in the form of quantitative information was collected using a questionnaire with closed-ended questions. The data collection period spanned from March 2023 to September 2023. Data collection tools were distributed to participants both in hand and in electronic form. The data collection tool was aligned with the study’s objectives.

Section A focused on demographic data, while Section B assessed the knowledge about CPD e-learning platforms using various scales: knowledge of action scale used 5-points, level of familiarity scale used 5-points, modes of participation scale used 3-points, and CPD platform scale used 7-points.

In Section C, the usability of CPD e-learning platforms was evaluated using items/scales described in the System Usability Scale [14]. Subsections (i) and (ii) assessed the easiness of using CPD e-learning platform and satisfaction related to its use respectively, ease of use scale used (5-points), frequency scale used (5-points), priority level scale used 5-points, agreement scale used 5-points, sufficiency scale used 5-points, satisfaction rating scale used 5-points. Subsection (iii) evaluated the efficiency of CPD e-learning platform and the frequency rating scale used (5-points, level of problem scale used 4-points, agreement scale used 5-points. Subsection (iv) evaluated the intuitiveness of CPD e-learning platform: level of clarity scale used 5-points, level of accessibility scale used 5-points, and the agreement scale used 5-points.

The subsection (v) assessed the usefulness/importance of CPD e-learning platforms: the agreement scale used 5-points, and the level of importance scale also used 5-points.

Section D focused on anticipated ICT technical issues and other challenges using scales such as the agreement scale with 5-points, the level of problem scale with 4-points, the level of accessibility scale with 5-points, the level of familiarity scale using 5-points, along with a Yes/No Scale which used 2-points. Using such scales facilitated data collection from large samples over a short period and provided insights for analysis and interpretation. The tool’s closed-ended questions made the data analysis easier and the data administration more efficient.

### Data analysis

The quantitative data was entered into the IBM SPSS Statistics 25 software (Statistical Package for the Social Sciences, SPSS) for further analysis. To ensure the validity and reliability of our data, we assessed construct validity and reliability. Descriptive statistics were used to provide an overview of the data. We calculated frequencies and percentages for categorical variables, such as healthcare provider’s knowledge about CPD platforms, the usability of e-learning platforms, and the challenges they faced.

### Constructs validity

The Table 1 above shows that all the constructs in question achieved loading factors exceeding 50%, indicating a strong validation of the model. To enhance the validity of the measured constructs even further, the researcher conducted a pre-test of the survey questionnaire and this was also complemented by interviews with the study participants. This approach confirmed significant correlations and support for all the constructs used in the study, with convergent validity values greater than zero.

**Table 1:** Constructs validity

Constructs	Initial	Factors loading
Knowledge about CPD platforms Usability of CPD platforms Challenges to use CPD platforms	1.000 1.000 1.000	0.715 0.717 0.507

### Construct’s reliability

The reliability of the constructs in this study was measured on each item by using the Cronbach’s Alpha [15] As reported in Table 2, the reliability analysis indicates that all the constructs used in the study questionnaire explain a positive degree of construct reliability.

**Table 2:** Constructs reliability

Constructs	Cronbach’s alpha	Number of items
Knowledge about CPD platforms Usability of CPD platforms Challenges to use CPD platforms	0.495 0.813 0.337	5 25 10

Based on the conducted reliability analysis all the items proposed under each construct have been used to collect data for this study.

## Results

In this study, 140 out of 240 participants returned a completed questionnaire, resulting in a participation rate of 58.3%. The results indicate that the majority of participants are females 71% (99/140) as indicated in Table 3:

**Table 3:** Participants gender

Gender				
	Frequency	Percent	Valid percent	Cumulative percent
Male	41	29.3	29.3	29.3
Female	99	70.7	70.7	100.0
Total	140	100.0	100.0	

Accordingly, as indicated in Table 4, the majority of the participants belong to the category of nurses (63%) (88/140), while the rest were midwives (23%) and allied health professionals with 29% representation.

**Table 4:** Participants category

Category of staff				
	Frequency	Percent	Valid percent	Cumulative percent
Nurse	88	62.9	62.9	62.9
Midwife	23	16.4	16.4	79.3
Allied health professional	29	20.7	20.7	100.0
Total	140	100.0	100.0	

Regarding the participants’ level of education, the majority possess A_1_ and A_0_ degrees respectively, especially for general nursing and midwifery. Very few participants (2.9%) are master’s degree holders as reported in Table 5 below.

**Table 5:** Participants education level

Qualification				
	Frequency	Percent	Valid percent	Cumulative percent
General nursing A1	47	33.6	33.6	33.6
Midwifery A1	12	8.6	8.6	42.1
General nursing A0	38	27.1	27.1	69.3
Midwifery A0	10	7.1	7.1	76.4
Laboratory technician A1	3	2.1	2.1	78.6
Laboratory technician A0	7	5.0	5.0	83.6
Radiology technician A0	9	6.4	6.4	90.0
Physiotherapist A0	6	4.3	4.3	94.3
Pharmacist A0	2	1.4	1.4	95.7
Prosthetist and orthotist A0	2	1.4	1.4	97.1
Master’s degree	4	2.9	2.9	100.0
Total	140	100.0	100.0	

Moreover, several participants in this study have been in the healthcare profession for 10 years and more and a good number of them has this experience for more than 4 years. More information on experience in health profession is visualized in Table 6 below.

**Table 6:** Participants experience in health sector

Experience in health profession				
	Frequency	Percent	Valid percent	Cumulative percent
Experience 0–3 years	12	8.6	8.6	8.6
Experience 4–5 years	20	14.3	14.3	22.9
Experience 6–10 years	36	25.7	25.7	48.6
Above 10 years	72	51.4	51.4	100.0
Total	140	100.0	100.0	

Interestingly, the total number of participants 100% (140/140) reported that they are aware of and utilize CPD e-learning platforms during their courses.

### Participants familiarity with CPD e-learning platforms

Regarding their familiarity with the e-learning platforms, 4.3% (6/140) of the participants are not at all familiar with it, while 95.7% (134/140) reported being familiar. When it comes to the mode of accessing courses, 82.1% (115/140) of the participants took their courses through online, while 12.9% (18/140) attended courses on-site and 5% (7/140) utilized video conferencing. Regarding the Rwandan CPD e-learning platforms, the majority of participants 71.4% (100/140) used National Council of Nurses and Midwives of Rwanda (NCNMR) CPD platform, surpassing other Rwandan CPD providers.

The study also revealed that the primary purpose of using e-learning platforms was to renew their licenses, as indicated by 71.4% (100/140) of the participants as shown in Table 7.

**Table 7:** Knowledge about CPD through e-learning platforms (*n* = 140)

Variable		Frequency(*n*)	Percentage (%)
Have you heard about CPD e-learning platform?	Never	0	0
	Rarely	8	5.7
	Sometimes	25	17.9
	Often	52	37.1
	Always	55	39.3
Have you ever used CPD e-learning platform?	Never	0	0
	Rarely	22	15.7
	Sometimes	51	36.4
	Often	38	27.1
	Always	29	20.7
Are you familiar with the CPD e-learning platform?	Not at all familiar	6	4.3
	Slightly familiar	16	11.4
	Somewhat familiar	27	19.3
	Moderately familiar	59	42.1
	Extremely familiar	32	22.9
How do you get CPD courses?	Online	115	82.1
	Onsite	18	12.9
	Video conferences/meeting	7	5.0
Which Rwandan CPD provider company or organization do you use to attend online CPD courses?	Rwanda Biomedical Center (RBC) CPD platform	6	4.3
	Ministry of Health CPD platform	12	8.6
	Allied Health Professions Council (RAHPC) CPD platform	7	5.0
	National Council of Nurses and Midwives Rwanda (NCNMR) CPD platform	100	71.4
	Rwanda Pharmaceutical Association (RPhA) CPD platform	2	1.4
	University of Rwanda CPD platform	2	1.4
	Other	11	7.9
Why did you choose/ or use e-learning for CPD?	To renew my license	100	71.4
	To improve my knowledge	33	23.6
	Required by the hospital	6	4.3
	To improve my ICT skills	1	0.7

**Fig. 2:**
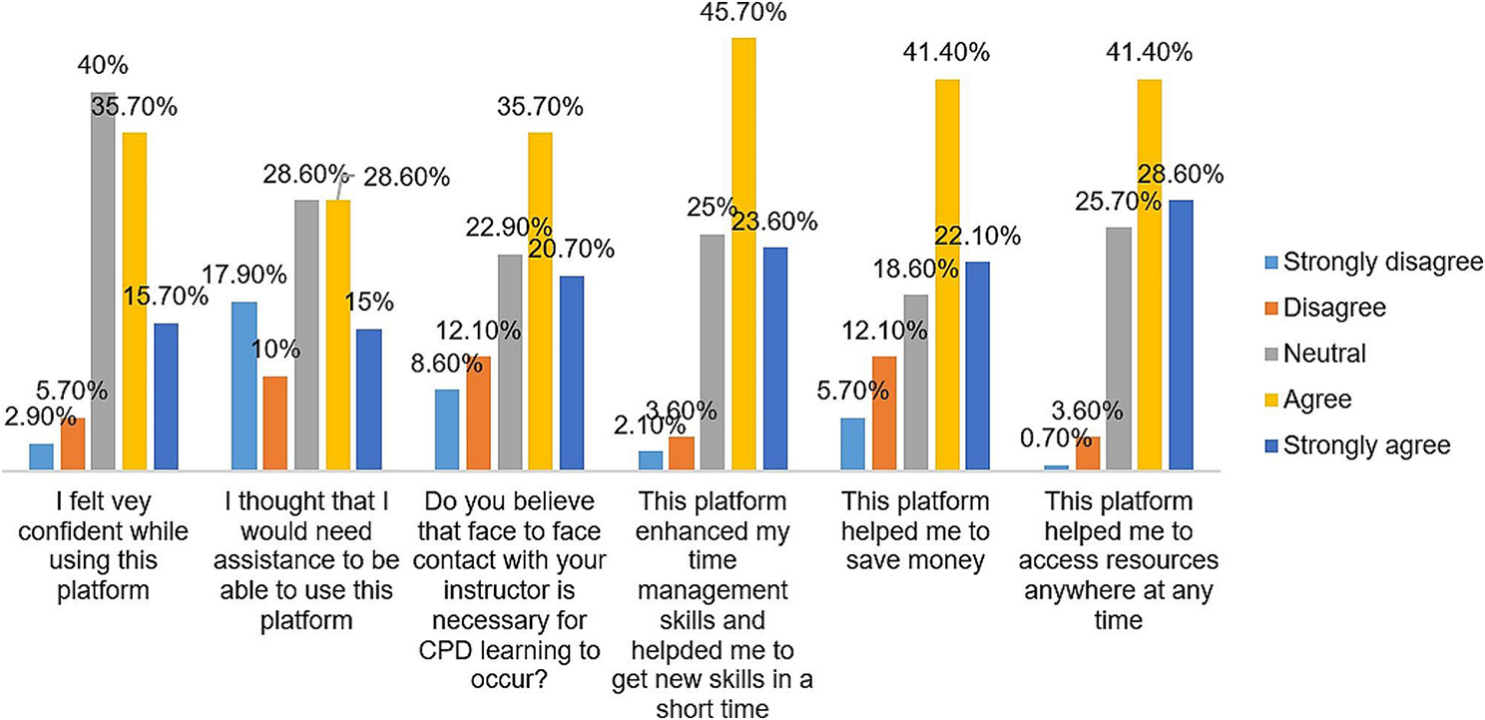
Six indicators of participants’ perception on CPD e-learning usability each expressed in terms of frequency of agreement or disagreement

Figure 2 below summarizes the finding about six indicators of usability of CPD e-learning platforms. It indicates that, 40% (56/140) had a neutral option, 35.7% (50/140) agreed and 15.7% (22/140) strongly agreed that they felt very confident when using the platform. Next, it indicates that 28.6% (40/140) had a neutral option, 28.6% (40/140) agreed and 15% (21/140) strongly agreed that they needed assistance to use the CPD e-learning platform.

Regarding the necessity of face to face with the instructor, 35.7% (50/140) agreed and 20.7% (29/140) strongly agreed that face-to-face with the instructor is necessary for CPD learning to occur. We also found that 45.7% (64/140) agreed and 23.6% (33/140) strongly agreed that the CPD e-learning platform improved their time management skills and helped them acquire new skills in a short time. In addition, 41.4% (58/140) agreed and 22.1% (31/140) strongly agreed that the CPD e-learning platform helped them to save money.

Finally, a large number of participants reported that the platform helped them to access CPD resources anywhere and anytime, with 41.4% (58/140) agreeing and 28.6% (40/140) strongly agreeing.

**Fig. 3:**
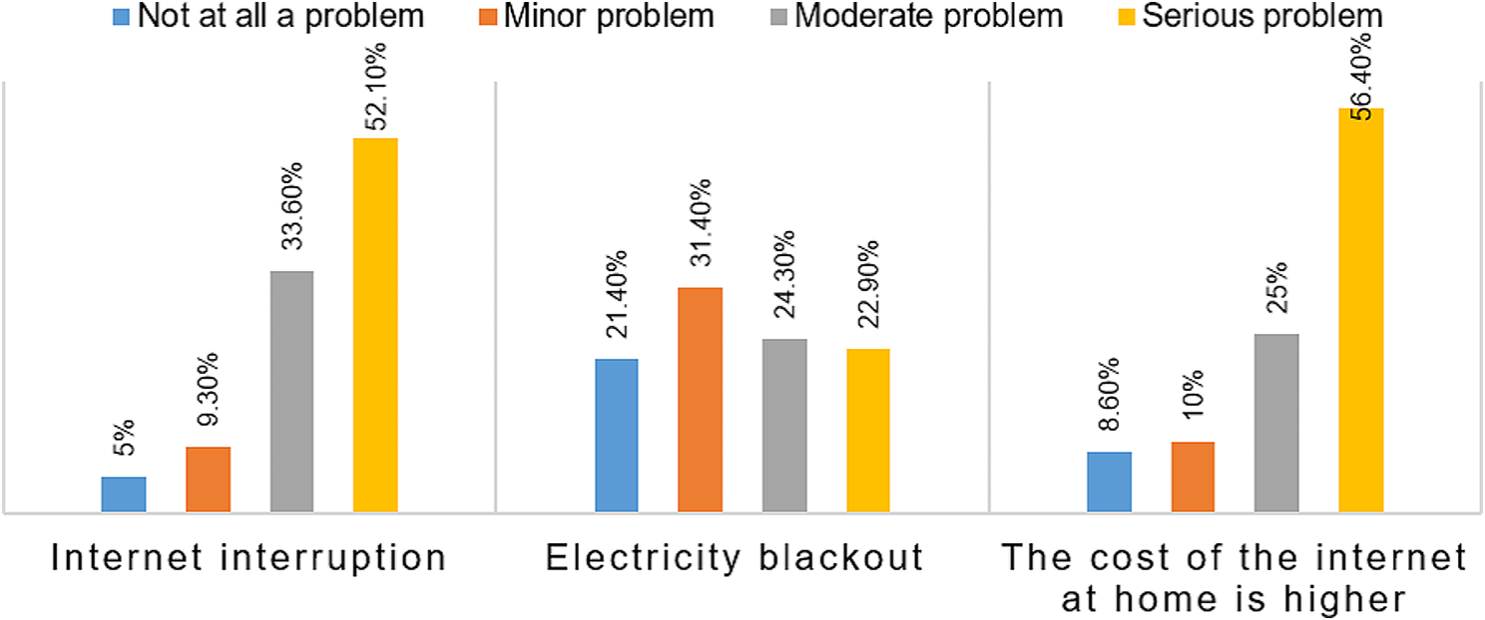
Results of evaluation of anticipated ICT technical issues and other challenges

Results about challenges of using e-learning based CPD courses are shown in Fig. 3 below. In summary, 33.6% (47/140) of the participants reported internet interruption as a moderate problem, and 52.1% (73/140) reported it as a serious problem while using the CPD e-learning platform. Consequently, the majority of participants encountered internet interruptions while using the CPD e-learning platform.

Furthermore, 31.4% (44/140) of the participants reported electricity blackout as a minor problem, 24.3% (34/140) reported it as a moderate problem, and 22.9% (32/140) reported it as a serious problem. Consequently, the majority of participants experience electricity blackout as a challenge when using this platform.

Accordingly, from the Fig. 3 below, it can be observed that participants reported the higher cost of internet as a minor problem by 10% (14/140), a moderate problem by 25% (35/140), and a serious problem by 56.4% (79/140) when using the CPD e-learning platform at home.

## Discussion

This study explored the usability of e-learning platforms for CPD of healthcare professionals. Regarding the easiness of the used e-learning platforms, the majority of participants (50.7%) expressed that the CPD e-learning platforms were easy to use, indicating a positive perception of its usability. However, a significant portion (22.1%) disagreed on its easiness, suggesting areas that may require improvement. Additionally, a considerable percentage (43.6%) required assistance while using the e-learning platforms during their CPD courses, indicating potential usability issues or complexities that users encountered. Regarding the e-learning platforms efficiency, the findings indicate that while a notable percentage (37.1%) use the e-learning platforms frequently, there is also a considerable proportion (45.7%) who are neutral about its efficiency. Understanding the factors contributing to this neutrality can provide insights into areas where the platform can be optimized to enhance efficiency and productivity for users.

The majority of participants (77.9%) in this study agreed that all multimedia elements (images, sound, text) in the e-learning platform were clearly visible and accessible, indicating positive feedback regarding the platform’s design and presentation. Hence, this explains a considerable level of the platform intuitiveness vis-à-vis the healthcare professionals. However, the presence of disagreement (18.6%) suggests that there may be aspects of the e-learning platform’s intuitiveness that require attention. Additionally, the perception of lack of interaction or discussion with others as a threat to course completion highlights the importance of fostering engagement and collaboration within the e-learning environment that are used for CPD of healthcare professionals.

Furthermore, while nearly half of the participants (49.3%) expressed satisfaction with the e-learning platform for their learning needs during their CPD, a significant proportion (32.1%) reported dissatisfaction. This underscores the importance of addressing user concerns and preferences to improve overall satisfaction levels. Furthermore, comparing CPD e-learning versus face-to-face courses, a majority of participants (60%) expressed satisfaction, indicating the perceived value and effectiveness of the e-learning approach for CPD. However, the presence of dissatisfaction (22.1%) suggests areas where traditional face-to-face methods may still hold advantages for some users. With these findings, a blended learning mode for CPD could be promoted to cater for both learning needs and preferences of the healthcare professionals in the case study institution.

Overall, regarding the usability of CPD e-learning platform, healthcare professionals exhibited a high level of confidence (51.4%) when utilizing CPD e-learning platforms, which aligns with the findings supported by the literature review [4]. Concerning the need for assistance in using CPD e-learning platforms, the results indicate that 43.6% of participants recognized the necessity for training.

While e-learning offers numerous benefits for healthcare professionals, the results also underscore the importance of face-to-face instruction, particularly for practical skills as 56.40% agreed that face to face with instructor is necessary for e-learning. As for time management and the ability to quickly acquire new skills, the majority (69.3%) of healthcare professionals reported that they were able to learn new skills in a short period. The majority of participants (63.5%) acknowledged the cost savings and convenient access to resources provided by the platform.

These findings align with previous research on e-learning, emphasizing the advantages of online platforms in generating student interest, enhancing learning outcomes, and streamlining teaching practices [16]. Cost effectiveness, time saving, accessibility of resources anytime and anywhere are reported as an e-learning benefits [11]. Online CPD opportunities were well-received by healthcare workers in sub-Saharan Africa from diverse backgrounds. The study conducted by Feldacker et al. [4] suggested the need to expand these opportunities to provide more flexibility for self-directed learning. However, it is important for such courses to take into account the limited resources of individuals seeking these educational opportunities. The findings of Cheok et al. [17] reveal that students’ experience, or lack thereof, does not carry significant weight in this study. However, it does confirm that usability attributes are crucial for facilitating natural and spontaneous interactions with e-learning websites.

Regarding time savings, most healthcare professionals emphasized that CPD e-learning platforms helped them save on transportation expenses. In terms of resource accessibility at any time and from anywhere, the results demonstrated that the majority of participants were able to access platform resources whenever and wherever they needed.

These findings are supported by the existing literature which reported that the cost effectiveness, time saving, accessibility of resources anytime and anywhere are reported as an e-learning benefits [11, 18].

Despite this usability of E-learning based platforms, there are also challenges related to them; the majority of participants (77.9%) did not have their own computers, and sometimes used their smartphones to access the platform. Familiarity with the instructional language (English) was a limitation for some participants, with only 15.7% being familiar. Providing additional language options for users is recommended. Participants reported internet interruptions while learning, with 52.10% experiencing them. Additionally, only 40% felt they had sufficient time to attend CPD courses, emphasizing the need for additional time provided by the hospital in the workplace.

Accordingly, 56.40% of participants reported a high home internet cost, suggesting a need for reduced internet costs. These findings are supported by findings from previously study which reported that the absence of internet access and computers at home for students, particularly in rural areas, hindered their involvement in e-learning beyond the classroom [16, 19]. Highlighting the importance of technical support, training, and infrastructure improvement to enhance e-learning experiences. Therefore, It is crucial to bridge the digital divide, promote a culture of shared learning, and provide training to effectively use e-learning systems [20].

## Conclusions

This study evaluated the knowledge, usability, and challenges of CPD e-learning platforms for healthcare professionals at CHUK. Participants expressed confidence in using the platforms but highlighted the need for further support and training. Factors such as time constraints, technical assistance, the high cost of internet, resource accessibility, and language proficiency were identified as important factors to consider for improving the e-learning based CPD experience. Implementing these findings can improve Rwandan CPD e-learning platforms dedicated to healthcare professionals.

In conclusion, the findings of this study highlight both strengths and areas for improvement in the CPD e-learning platform for healthcare professionals. By addressing e-learning platform usability issues, optimizing efficiency, enhancing intuitiveness, and aligning the platform with user satisfaction, developers and educators’ expectations, can create a more effective and engaging online learning experience for healthcare professionals pursuing continuing education. To mitigate some challenges for adopting CPD e-learning platforms by healthcare professionals, participants expressed the belief that every healthcare professional embarking on CPD should have their own laptop and that hospitals should provide a reliable internet connection during the training period. In alignment with the study by Cheok et al. [16] analyzing the teachers’ perceptions of e-learning in secondary schools, this study reported also that there is a need for additional training and technical support to improve health professional digital literacy skills and confidence in utilizing new technologies such as e-learning platforms.

More emphasis should also be put on the importance of the e-learning platform to accommodate different languages, and include interactive live questions and answers. Additionally, as new technologies for e-learning platforms are not conversant for several healthcare professionals, some face-to-face sessions with instructors for each course are also recommended, which in this case a blended learning mode for CPD.

## Data Availability

The datasets generated and analyzed during the current study are not publicly available due to a lack of ethical approval for sharing the raw data. However, they can be obtained from the corresponding author upon reasonable request.
